# Supplementary material for: Antimicrobial Efficacy of 7-Hydroxyflavone Derived from Amycolatopsis sp. HSN-02 and Its Biocontrol Potential on Cercospora Leaf Spot Disease in Tomato Plants
Source: Antibiotics (Basel). 2023 Jul 11;12(7):1175. doi: 10.3390/antibiotics12071175 (PMC10376496; doi:10.3390/antibiotics12071175)
Supplement: Supplementary file 1 [file antibiotics-12-01175-s001.zip › antibiotics-2427169-supplementary.pdf]

Antimicrobial Efficacy of 7-Hydroxyflavone Derived from *Amycolatopsis* sp. HSN-02 and Its Biocontrol Potential on Cercospora Leaf Spot Disease in Tomato Plants

Halaswamy Hire Math [1], Raju Suresh Kumar [2], Bidhayak Chakraborty [1], Abdulrahman I. Almansour [2], Karthikeyan Perumal [3], Girish Babu Kantli [4] and Sreenivasa Nayaka [1,*]

1 P.G. Department of Studies in Botany, Karnatak University, Dharwad 580003, Karnataka, India; hmhalaswamy2063@gmail.com (H.H.M.); pallabchakraborty3@gmail.com (B.C.)
2 Department of Chemistry, College of Science, King Saud University, P.O. Box 2455, Riyadh 11451, Saudi Arabia; sraju@ksu.edu.sa (R.S.K.); almansor@ksu.edu.sa (A.I.A.)
3 Department of Chemistry and Biochemistry, The Ohio State University, 151 W. Woodruff Ave, Columbus, OH 43210, USA; perumal.11@osu.edu
4 Department of Life Sciences, PIAS, Parul University, Vadodara 391760, Gujarat, India; gibugiri@gmail.com
* Correspondence: sreenivasankud@gmail.com

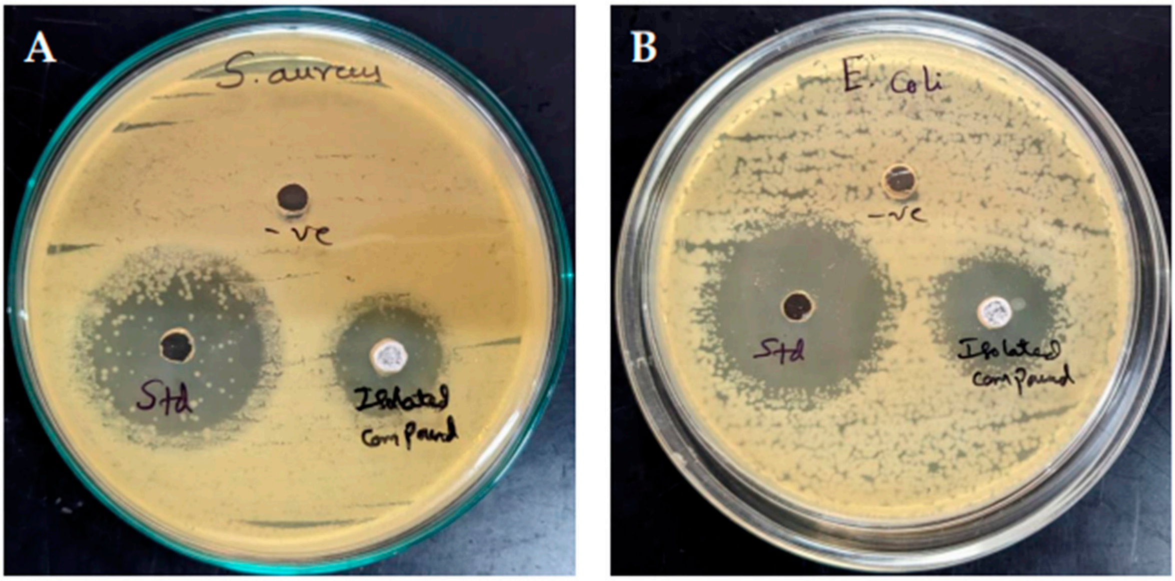

Figure S1. Antibacterial activity of standard drug and purified compound: (A) *S. aureus* and (B) *E. coli.*

Table S1. Detailed HRMS analysis of SC medium without the inoculation of *Amycolatopsis* sp. HSN-02.

Compound	Retention Time	Adduct Ion	*m/z* Value
1	7.88	$[M+H]^+$	219.1698
2	10.96	$[M+H]^+$	284.3261
3	11.36	$[M+H]^+$	149.0255
4	11.51	$[M+H]^+$	512.3919
5	11.87	$[M+H]^+$	540.4945
6	12.7	$[M+H]^+$	881.6713

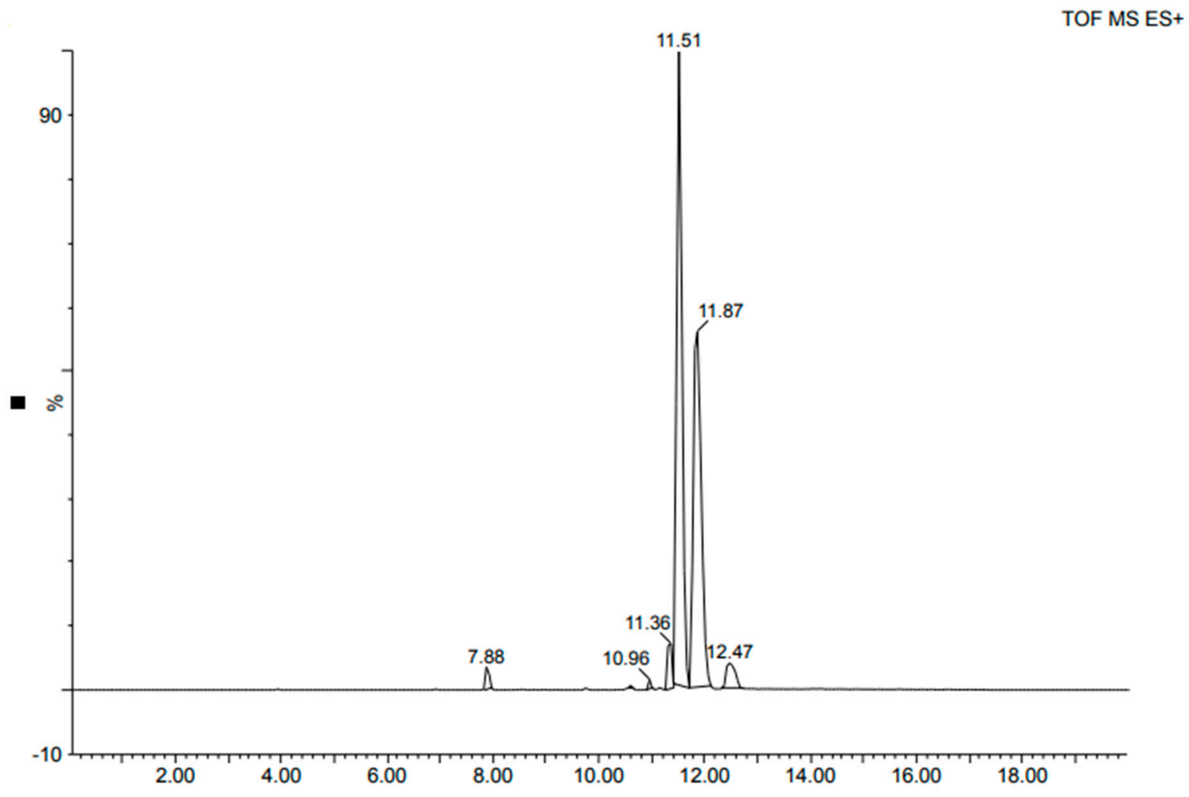

Figure S2. HRMS chromatogram of SC medium without the inoculation of *Amycolatopsis* sp. HSN-02.
